# CGRP-monoclonal antibodies in difficult-to-treat chronic migraine patients

**DOI:** 10.1007/s10072-022-06154-0

**Published:** 2022-05-26

**Authors:** Francesca Schiano di Cola, Salvatore Caratozzolo, Marco Bolchini, Giulia Ceccardi, Matteo Cortinovis, Paolo Liberini, Alessandro Padovani, Renata Rao

**Affiliations:** 1 Clinical and Experimental Sciences Department, Neurology Unit, University and SpedaliCivili, Brescia, Italy; 2 Clinical and Experimental Sciences, Università Degli Studi Di Brescia, ASST Spedali Civili Brescia, Piazzale Spedali Civili, 1, 25123 Brescia, Brescia (BS) Italy

**Keywords:** CGRP, Migraine, Erenumab, Prophylaxis, Disability

Migraine prevention has historically been characterised by poor tolerability and adherence of available oral drugs, with little efficacy in a considerable percentage of patients [1]. Prior to calcitonin gene–related peptide (CGRP)-monoclonal antibodies (mAbs), onabotulinumtoxinA (BoNTA) was the only specifically approved preventative medications in the USA and Europe for the prophylaxis of chronic migraine (CM). Randomised clinical trials extensively proved CGRP-mAb efficacy in episodic and chronic migraine, with real-life studies further confirming their efficacy also in ‘refractory’ patients with medication overuse [2, 3]. The aim of the present study was to assess CGRP-mAb efficacy in patients with a diagnosis of CM who previously failed or had contraindications to at least five different anti-migraine treatment classes (beta-blockers, antiepileptic drugs, tricyclic antidepressants, calcium channel blockers, onabotulinumtoxinA).

The present work is an observational retrospective study conducted at the Headache Centre – Spedali Civili Brescia from November 2020 to January 2022. The study included all adult patients with a diagnosis of CM in prophylactic treatment with a CGRP-mAb (erenumab, galcanezumab or fremanezumab) with an available 12-month follow-up. Inclusion criteria were as follows: documented history of migraine for at least 12 months, diagnosis of CM for at least 3 months prior to study enrolment, ≥ 5 previous prophylactic failures (beta-blockers, antiepileptic drugs, tricyclic antidepressants, calcium channel blockers, onabotulinumtoxinA). Patients were assessed at baseline (T0) and following 3 (T3), 6 (T6) and 12 (T12) months of treatment. Patients’ data regarding migraine history, clinical and demographical information, previous and current acute and preventive migraine treatments and concomitant medications were collected. Monthly headache and migraine days (MHDs and MMDs), analgesic consumption and attacks’ pain intensity (Numerical Rating Scale (NRS)) were also collected. Patients were asked to complete migraine disability questionnaires (HIT-6 and MIDAS) quarterly.

Fifty patients were enrolled, of whom 33 in treatment with erenumab 140 mg every 4 weeks, 15 with galcanezumab 120 mg monthly (following the first loading dose of 240 mg) and 2 with fremanezumab 225 mg monthly. All patients documented medication overuse. Clinical and demographical data of all patients are presented in Table 1. At T3, T6 and T12, respectively, 55.4%, 64.2% and 72.7% of patients documented a > 50% MHD reduction. Mean MHDs decreased from baseline 20.5 (SE 1.2) to 8.7 (SE 1.1) at T6 and to 7.6 (SE 0.9) at T12 (*p* < 0.0001). Mean MIDAS scores decreased from baseline 104.1 (SE 17.1) to 31.2 (SE 7.9) at T6 and to 17.7 (SE 5.6) at T12 (*p* = 0.004). Mean HIT-6 scores also improved from baseline 66.6 (SE 2.8) to 57.7 (SE 2.7) at T6 to 55.7 (SE 2.4) at T12 (*p* = 0.05). Monthly analgesic consumption decreased from baseline 20.9 (SE 1.5) to 10.3 (SE 1.5) at T6 and to 7.2 (SE 0.9) at T12 (*p* < 0.0001). Finally, the mean reported pain intensity (NRS scores) decreased from 7.5 (SE 0.1) at T0 to 6.2 (SE 0.3) at T6 and to 5.5 (SE 0.3) at T12 (*p* < 0.0001).

**Table 1:** Clinical and demographical characteristics of all patients

	Patients (*n* = 50)
Gender (female *n*, %)	42 (84%)
Age (mean, SD)	48.2 (9.1)
CM disease duration, years (mean, SD)	28.4 (10.1)
Previous prophylaxes (mean, SD)	6.6 (1.8)
Type of MO (*n*, %)	
NSAIDs	12 (24%)
Triptans	34 (68%)
Multiple drug classes	4 (8%)
Triptan responders (*n*, %)	36 (72%)
Relationship status (*n*, %)	
Single	13 (26%)
Stable relationship	35 (70%)
Not available/not given	2 (4%)
Night shifts (*n*, %)	12 (24%)
Comorbidities (*n*, %)	
Psychiatric	22 (44%)
Cardiovascular	25 (50%)
Endocrine	8 (16%)

*SD*, standard deviation; *CM*, chronic migraine; *MO*, medication overuse; *NSAIDs*, non-steroidal anti-inflammatory drugs

A further analysis was then carried out in order to compare the percentage of CGRP-mAb responders in our cohort of difficult-to-treat patients versus patients who only failed up to three previous prophylaxes. This second cohort comprised all patients currently in treatment with CGRP-mAbs with three previous treatment failures and a diagnosis of CM and MO, enrolled from November 2020 with a 12-month follow-up. This group comprised a smaller number of patients (*n* = 28) but was matched to our main cohort in terms of sex (89% female), age (mean age 47.7 years old) and disease duration (28.1 years). All patients were treated with erenumab 140 mg. No significant differences were found in the percentages of responders in the two groups at T3 (55.4% vs 55.6%, *p* = 0.6), T6 (64.2% vs 68.8%, *p* = 0.07) and T12 (72.7% vs 71.1%, *p* = 0.8).

Our preliminary results confirm previous reports regarding the efficacy of CGRP-monoclonal antibodies even in patients with ‘refractory’ chronic migraine and medication overuse who previously failed numerous preventive treatments [2, 3], including onabotulinumtoxinA. Such clinical improvement was sustained and progressive over time. Moreover, this effect did not seem to be influenced by the level of previous failures, as demonstrated by the fact that no differences could be found in terms of MHD reduction compared to our ‘not so difficult to treat’ migraine cohort (i.e. patients who failed up to a maximum of three previous migraine prevention treatments). The improvement in migraine frequency and intensity led to a significant reduction also in headache-related disability and analgesic consumption in our very complex patients. The present study has some limitations. Firstly, the number of patients was limited by the long follow-up. Secondly, the ‘not so difficult to treat’ group of patients was smaller and all patients were in treatment with the same CGRP-mAbs, and these could have biassed our results. Moreover, given the small sample no differences could be drawn in terms of efficacy between the different CGRP-mAbs. Further studies will be needed to evaluate whether disease duration or the number of previously failed prophylaxes might actually have an effect on the overall clinical response in patients treated with CGRP-monoclonal antibodies.
